# Social Capital and Mental Health in Japan: A Multilevel Analysis

**DOI:** 10.1371/journal.pone.0013214

**Published:** 2010-10-06

**Authors:** Tsuyoshi Hamano, Yoshikazu Fujisawa, Yu Ishida, S. V. Subramanian, Ichiro Kawachi, Kuninori Shiwaku

**Affiliations:** 1 Organisation for the Promotion of Project Research, Shimane University, Matsue, Japan; 2 Department of Environmental and Preventive Medicine, Shimane University School of Medicine, Izumo, Japan; 3 Division of Public Policy, School of Administration and Informatics, University of Shizuoka, Shizuoka, Japan; 4 Department of General Studies, Akashi National College of Technology, Akashi, Japan; 5 Department of Society, Human Development, and Health, Harvard School of Public Health, Boston, Massachusetts, United States of America; UCL Institute of Child Health, United Kingdom

## Abstract

**Background:**

A national cross-sectional survey was conducted in Japan. This is because the growing recognition of the social determinants of health has stimulated research on social capital and mental health. In recent years, systematic reviews have found that social capital may be a useful factor in the prevention of mental illness. Despite these studies, evidence on the association between social capital and mental health is limited as there have been few empirical discussions that adopt a multilevel framework to assess whether social capital at the ecological level is associated with individual mental health. The aim of this study was to use the multilevel approach to investigate the association between neighborhood social capital and mental health after taking into account potential individual confounders.

**Methodology/Principal Findings:**

We conducted a multilevel analysis on 5,956 individuals nested within 199 neighborhoods. The outcome variable of self-reported mental health was measured by the one dimension of SF-36 and was summed to calculate a score ranging from 0 to 100. This study showed that high levels of cognitive social capital, measured by trust (regression coefficient = 9.56), and high levels of structural social capital, measured by membership in sports, recreation, hobby, or cultural groups (regression coefficient = 8.72), were associated with better mental health after adjusting for age, sex, household income, and educational attainment. Furthermore, after adjusting for social capital perceptions at the individual level, we found that the association between social capital and mental health also remained.

**Conclusions/Significance:**

Our findings suggest that both cognitive and structural social capital at the ecological level may influence mental health, even after adjusting for individual potential confounders including social capital perceptions. Promoting social capital may contribute to enhancing the mental health of the Japanese.

## Introduction

Growing recognition of the social determinants of health has stimulated research on social capital and mental health. In recent years, systematic reviews using cross-sectional data have found that social capital may be a useful factor in the prevention of mental illness [1], [2]. In addition, a prospective study by Fujiwara and Kawachi [3] showed that a low level of social capital, measured by trust, is associated with depression.

As for measuring social capital, these previous studies have provided evidence for the importance of distinguishing the structural components of social capital (structural social capital) from its cognitive components (cognitive social capital). Structural social capital refers to what people do (e.g., participation in associations) while cognitive social capital refers to what people feel (e.g., trust in others, reciprocity between individuals) [4]. These two components may differentially affect mental health outcomes. For instance, a systematic review by De Silva [2] found that although cognitive social capital is associated with better mental health, structural social capital is associated with poor mental health.

Despite these previous studies, evidence on the association between social capital and mental health is limited by the fact that there has been little empirical research that adopts a multilevel framework to assess whether social capital at the ecological level is associated with individual mental health. That is, a multilevel model implies that variations in mental health outcomes are determined by compositional effects (e.g., age, sex, educational attainment, income) as well as by contextual effects such as community social capital [5]. Social capital at the ecological level has most often been measured by aggregating individual perceptions of social capital. However, the effect of social capital at the ecological level on mental health might be confounded by individual perceptions of social capital. We therefore need to understand whether social capital at the ecological level exerts a contextual effect on mental health, even after adjusting for individual perceptions of social capital.

In the present study, we defined community social capital based on the “cho-cho” or “aza” unit, which is made up of approximately 250 households. The aim of this study was to examine (1) whether cognitive and structural social capital at the ecological level were associated with individual mental health by means of a multilevel analysis; (2) whether social capital at the ecological level exert a contextual effect on mental health after individual perceptions of social capital were taken into account.

## Methods

### Data

Our data came from a cross-sectional survey conducted in March 2008. This survey aimed to determine social factors related to health, such as self-rated health and mental health. An anonymous self-administrated questionnaire which included mainly items on socioeconomic status, social capital and health outcome was designed. We conducted a nationally representative survey covering households across Japan as a whole. We used a “geodemographic segmentation system” as our sampling frame. The geodemographic segmentation system classifies households in Japan by allocating them to one of 212 segments at the small-area unit level (a cho-cho or aza unit level). In the present study, we defined a cho-cho or aza unit as a neighborhood; and each neighborhood was randomly selected from within each segment (1 neighborhood×212 segments = 212 neighborhoods). The survey eventually targeted 81,974 households on the basis of the National Census, and a postal questionnaire was sent out to all the heads of households and their spouses (a total of 120,846 individuals). A total of 8221 subjects (3937 males (47.9%), 4148 females (50.5%), and 136 subjects (1.7%) did not answer the gender question) were responded to the survey. The overall response rate was 6.8%.

The geodemographic segmentation system has been used in health-related research [6], [7] and in recent years has been adapted for social capital research [8], [9]. One reason for utilizing this system was that it captures well-defined units in a small area. Although there have been many studies on social capital and health, their evidence was dependent on the targeted areas [10]–[14]. This study therefore focuses on social capital in geographically defined neighborhoods.

### Measures

Social capital has been broadly defined as the features of social organization, such as trust, norms, and networks that can improve the efficiency of society by facilitating coordinated actions [15]. Here, we followed the cognitive and structural distinction to select the measures of social capital [4]. Cognitive social capital was measured by trust using a single item [4], [16]. The respondents were asked the following question: “Would you say that people in your neighborhood can be trusted or that you need to be very careful in dealing with them?” This question was rated on a 10-point scale, with 1 being excellent, and 9 being very poor, as well as “do not know.” We then dichotomized the responses. That is, the response “do not know” was excluded and ratings of 1–4 and 5–9 were collapsed into high trust and low trust, respectively. To consider the contextual effect of cognitive social capital, we aggregated individual response to the neighborhood level [5]. That is, we calculated the percentage of respondents who responded “high trust” for each neighborhood.

Structural social capital was assessed by the number of civic associations to which respondents belonged [4]. The respondents were required to state their affiliation to two different types of associations: neighborhood associations and sports, hobby, recreation, or cultural groups. Structural social capital was coded 0 for “No, I don't belong” and 1 for “Yes, I belong.” To consider the contextual effect of structural social capital, we aggregated individual response to the neighborhood level [5]. That is, we calculated the percentage of respondents who responded “Yes” for each neighborhood.

The outcome variable of self-reported mental health was measured by the SF-36 [17]. The SF-36 has been translated into Japanese and provides an empirical test of validity [18]. The SF-36 is based on eight dimensions: Physical functioning, Role physical, Bodily pain, Social functioning, General health perceptions, Vitality, Role emotional, and Mental health. We used the mental health dimension in this study, which included the following five items: “Have you been very nervous?”; “Have you felt so down in the dumps that nothing could cheer you up?”; “Have you felt calm and peaceful?”; “Have you felt downhearted and depressed?”; “Have you been happy?” All these items were rated on a 5-point Likert scale (“all of the time,” “most of the time,” “some of the time,” “a little of the time,” and “none of the time”) and were summed to calculate a score ranging from 0 to 100, with higher scores denoting better mental health. At the individual level, the internal consistency reliability (Cronbach's α) for this scale was 0.846.

In addition, we considered demographic and socioeconomic variables that were included in the survey as control variables. These variables included age, sex, educational attainment (ranging from primary school to college graduate), and annual household income (ranging from 0 to 12,000,000 yen). Household income was collapsed into the following five on the basis of the statistics of the Comprehensive Survey of Living Conditions of the People on Health and Welfare, which was conducted by the Ministry of Health, Labour and Welfare in Japan: (i) less than 2.0 million yen; (ii) 2.0 million yen −4.0 million yen; (iii) 4.0 million yen −6.0 million yen; (iv) 6.0 million yen −8.0 million yen; (v) more than 10 million yen.

### Analyses

After excluding missing data on the outcome, independent variables such as mental health, age, sex, educational attainment, household income, and social capital perceptions, we conducted a multilevel analysis on 5956 individuals nested within 199 neighborhoods (Table 1). In other words, 5956 individuals (at level 1) nested within 199 communities (at level 2) comprised the multilevel data structure considered in this analysis. Multilevel analysis offers a comprehensive framework for understanding the ways in which places can affect people (contextual effect), or people can affect places (composition) [5]. In the context of the analysis presented here, the multilevel analysis allows for estimation of (1) the overall associations between compositional factors and mental health (“fixed parameters”); (2) the effect of contextual factors, measured by trust, neighborhood associations and sports, hobby, recreation, or cultural groups, on mental health (“fixed parameters”); and (3) the variation in mental health between neighborhoods (“random parameters”). More specifically, we tested four sets of multilevel regression models (random intercept models). The following are detailed descriptions of the four models:

**Table 1:** Descriptive statistics for compositional and contextual variables used in models.

	n	Mean/%	SD	Range
**Compositional factors**				
Mental health	5956	65.1	18.3	0.0–100.0
Sex				
Male	3020	50.7		
Female	2936	49.3		
Age				
−30	353	5.9		
30–39	722	12.1		
40–49	796	13.4		
50–59	1118	18.8		
60–69	1686	29.3		
70–79	1052	17.7		
80–	229	3.8		
Household income (Yen million)				
<2.0	832	14.0		
2.0–4.0	1816	30.5		
4.0–6.0	1323	22.2		
6.0–8.0	820	13.8		
>8.0	1165	19.6		
Educational attainment				
Secondary school	720	12.1		
High school	2555	42.9		
Two-year college	885	14.9		
University	1608	27.0		
Graduate school	188	3.2		
Social capital perception				
Trust				
Low	2583	43.4		
High	3373	56.6		
Neighborhood associations				
No	2992	50.2		
Yes	2964	49.8		
Sports, Recreation, Hobby, Culture				
No	3808	63.9		
Yes	2148	36.1		
**Contextual factors**				
Social capital				
Trust	199	56.6	12.7	0.0–100.0
Neighborhood associations	199	49.7	17.0	0.0–100.0
Sports, Recreation, Hobby, Culture	199	36.0	10.1	0.0–100.0

Model 1: This is a two-level null (empty) model of individuals nested within neighborhoods (level 2) with only the constant term in the fixed and random parts. Variation in mental health was partitioned across individuals (within neighborhoods) and between neighborhoods. The intra-class correlation coefficient indicates the proportion of total variance that resides between neighborhoods at level 2 [19].

Model 2: This is the same as Model 1 with the added variables of compositional factors (age, sex, household income, educational attainment) in the fixed part. The model assessed the compositional effect on mental health.

Model 3: This is the same as Model 2 with the added variables of social capital. Here, the model assessed the contextual effect of social capital on mental health after adjusting for compositional factors.

Model 4: This is the same as Model 3 with the added variables of perceptions of social capital. We considered whether social capital at the neighborhood level exerted a contextual effect on mental health after adjusting for individual perceptions of social capital and other compositional factors. Since social capital at the neighborhood level was composed of aggregated individual responses, the contextual effect of social capital on mental health may be confounded by social capital perceptions. The purpose of this model was to examine whether the influence of social capital variables was truly contextual [20].

All statistical analyses were performed using the MLwiN software package (version 2.10).

### Ethics

The survey was completely anonymous and participation was voluntary. In addition, this study used data with no identifiable information on the survey participants. According to the ethical guidelines for epidemiological study by the Japanese government, written or verbal informed consent was not required for this type of study: the response to the survey constituted the participants' informed consent. The informed consent script provided information about the voluntary nature of the survey, content of the survey, and expected duration for the participants. Since our study design was not experimental and comprised no interventions, a formal ethical review of this study was not sought before conducting the survey.

## Results

Table 1 provides a summary of the data for the analysis. The mean score of mental health was 65.1 (standard deviation: 18.3), ranging from 0.0 to 100.0. Regarding demographic characteristics, close to half of the sample were male (50.7%), and 29.3% were 60–69 years of age, followed by 50–59 years of age (18.8%), and 70–79 years of age (17.7%). As for socioeconomic characteristics, 42.9% were high school graduates, and about 30.0% had more than university-level of education. 19.6% had household incomes of over 8 million yen and 14.0% had incomes under 2 million yen. In terms of social capital at neighborhood level, the mean percentage of reporting high trust was 56.6% (standard deviation: 12.7). The mean percentage of respondents active in neighborhood associations was 49.7% (standard deviation: 17.0); in sports, hobby, recreation, or cultural groups was 36.0% (standard deviation: 10.1).

Table 2 provides the results of the multilevel analyses. The null model with no predictors (Model 1) revealed a significant variation in mental health between neighborhoods (σ^2^_u0_ = 4.669). However, this result did not take into account the compositional characteristics. In Model 2, females were more likely to have lower mental health scores. For the age variables, respondents 50–59 years of age or older were more likely to have higher mental health scores. As for the socioeconomic variables, those having an income of 2.0 million yen or more were likely to have higher mental health scores. Further, those with educational attainments equal to or higher than high school education were more likely to have higher mental health scores.

**Table 2:** Fixed and random part results for the multilevel analytical models.^a^

	Model 1		Model 2		Model 3A		Model 3B		Model 3C	
Constant	65.224		55.454		50.403		54.332		52.770	
**Compositional factors**										
Sex			−1.003	(0.042)	−1,012	(0.040)	−0.972	(0.049)	−1.022	(0.038)
Age										
30–39			0.174	(0.884)	0.140	(0.905)	0.110	(0.928)	−0.004	(0.999)
40–49			−0.783	(0.515)	−0.951	(0.429)	−0.946	(0.434)	−1.000	(0.406)
50–59			2.620	(0.025)	2.338	(0.045)	2.437	(0.038)	2.296	(0.050)
60–69			6.808	(<0.001)	6.485	(<0.001)	6.626	(<0.001)	6.423	(<0.001)
70–79			7.491	(<0.001)	7.152	(<0.001)	7.331	(<0.001)	7.142	(<0.001)
80-			6.724	(<0.001)	6.213	(<0.001)	6.584	(<0.001)	6.428	(<0.001)
Household income										
(Yen million)										
2.0–4.0			3.038	(<0.001)	2.925	(<0.001)	3.000	(<0.001)	2.899	(<0.001)
4.0–6.0			5.248	(<0.001)	5.031	(<0.001)	5.200	(<0.001)	5.113	(<0.001)
6.0–8.0			6.135	(<0.001)	5.941	(<0.001)	6.092	(<0.001)	5.985	(<0.001)
>8.0			6.062	(<0.001)	5.819	(<0.001)	6.048	(<0.001)	5.919	(<0.001)
Educational attainment										
High school			2.160	(0.005)	2.206	(0.004)	2.216	(0.004)	2.113	(0.006)
Two-year college			2.654	(0.005)	2.666	(0.005)	2.737	(0.004)	2.616	(0.006)
University			2.941	(<0.001)	2.925	(<0.001)	3.108	(<0.001)	2.878	(0.001)
Graduate school			3.910	(0.012)	4.072	(0.009)	4.162	(0.008)	3.820	(0.014)
**Contextual factors**										
Social capital										
Trust					9.565	(<0.001)				
Neighborhood associations							2.381	(0.132)		
Sports, Recreation, Hobby, Culture									8.726	(<0.001)
**Random parameters**										
Between neighborhoods b	4.669(1.587)		2.119(1.143)		1.123(0.952)		1.994(1.120)		1.150(0.960)	
Intra-class correlation	0.013		0.006		0.003		0.006		0.003	

a Regression coefficient reported and p-value in parentheses,

b SE in parentheses.

In Model 3, we observed that higher scores of cognitive social capital, measured by trust, as well as structural social capital, measured by membership in sports, recreation, hobby, or cultural groups, were more likely to have higher mental health scores, after adjustment for individual confounders (Table 2, Models 3A and 3C). Finally, after adjusting for social capital perceptions at the individual level, we found that the association between neighborhood social capital and mental health also remained (Table 3, Models 4A and 4C).

**Table 3:** Fixed and random part results for the multilevel analytical models.^a^

	Model 4A		Model 4B		Model 4C	
Constant	51.534		55.040		53.718	
**Compositional factors**						
Sex	−0.954	(0.051)	−1.007	(0.042)	−1.329	(0.007)
Age						
30–39	0.129	(0.912)	−0.119	(0.920)	0.036	(0.974)
40–49	−1.414	(0.234)	−1.278	(0.291)	−1.138	(0.343)
50–59	1.624	(0.160)	1.879	(0.112)	1.970	(0.091)
60–69	5.507	(<0.001)	5.882	(<0.001)	5.656	(<0.001)
70–79	6.038	(<0.001)	6.470	(<0.001)	6.412	(<0.001)
80-	4.906	(0.001)	5.653	(<0.001)	5.821	(<0.001)
Household income						
(Yen million)						
2.0–4.0	2.569	(<0.001)	2.871	(<0.001)	2.807	(<0.001)
4.0–6.0	4.480	(<0.001)	5.054	(<0.001)	4.945	(<0.001)
6.0–8.0	5.127	(<0.001)	5.866	(<0.001)	5.782	(<0.001)
>8.0	5.045	(<0.001)	5.782	(<0.001)	5.686	(<0.001)
Educational attainment						
High school	2.131	(0.005)	2.126	(0.006)	1.789	(0.021)
Two-year college	2.248	(0.017)	2.629	(0.006)	2.222	(0.020)
University	2.544	(0.003)	2.993	(0.001)	2.373	(0.007)
Graduate school	3.450	(0.025)	4.126	(0.008)	3.568	(0.022)
Social capital perception						
Trust	5.567	(<0.001)				
Neighborhood associations			2.003	(<0.001)		
Sports, Recreation, Hobby, Culture					3.183	(<0.001)
**Contextual factors**						
Social capital						
Trust	4.487	(0.023)				
Neighborhood associations			0.634	(0.700)		
Sports, Recreation, Hobby, Culture					5.909	(0.017)
**Random parameters**						
Between neighborhoods b	1.368 (0.985)		2.092 (1.136)		1.228 (0.970)	
Intra-class correlation	0.004		0.006		0.003	

a Regression coefficient reported and p-value in parentheses,

b SE in parentheses.

## Discussion

This study suggests that cognitive social capital as well as structural social capital, measured by membership in sports, recreation, hobby, or cultural groups, were associated with individual mental health status (Models 3A and 3C). Specifically, we focused on social capital at the ecological level in a geographically defined neighborhood, and our findings advance the empirical evidence on social capital and mental health within small area units. In addition, our findings enabled us to answer the question: do neighborhood levels of social capital exert a contextual effect on mental health after adjustment for perceptions of social capital? We found that both cognitive social capital and structural social capital appeared to have a contextual influence on mental health in Japan (Models 4A and 4C). Furthermore, since we developed the statistical models sequentially, we could examine changes in the amount of neighborhood variance in mental health explained by the variables in the regression. For example, comparing Models 1 and 3A, the neighborhood variances were reduced. This result suggested that the variation found by Model 1 was explained by differences in the compositional factors and contextual factors.

Our findings are consistent with some previous individual-level and ecological-level studies on social capital and mental health. For example, the literature review by De Silva et al. [2] reported that cognitive social capital is associated with better mental health. In addition, a multilevel study in a rural population found that cognitive social capital, measured by trust was, positively associated with psychological health [21]. On the other hand, Stafford et al. [22] reported that there was no evidence of an association between cognitive social capital, measured by trust, and mental health based on multilevel analyses in the United Kingdom. Thus, these empirical findings implied that further comparative studies were needed to investigate the main effects of social capital, measured by trust, on mental health for the sake of drawing robust conclusions.

Other components of social capital, measured by membership in sports, hobby, recreation, or cultural groups, showed a statistically significant association with mental health (Model 3C). On the other hand, structural social capital, measured by membership in neighborhood associations, was not significant (Model 3B). The results of the systematic reviews showed that structural social capital, although associated with better mental health, was sometimes found to be associated with poorer mental health [2]. A possible explanation for this different statistical result between Models 3B and 3C could be that people who belong to neighborhood associations sometimes may feel burdened or suffer from a sense of duty, such as the need to attend meetings regularly or to assume managerial or supervisory roles for neighborhood-based projects. Indeed, in order to lessen the burden, neighborhood associations in recent years have often selected members from different households based on a rotating system to serve in a supervisory capacity. Thus membership in such associations is less voluntary and members might experience fewer benefits than would those who voluntarily decide to become members of sports, hobby, recreation, or cultural groups. A previous study by Kondo et al. [23] also reported that these kinds of non-cohesive community activities may be harmful to the health of the members. This hypothesis, of course, requires further in-depth investigation.

Our study had several limitations. First, the present study used a cross-sectional design, so that we could not establish the temporal order of causality. In other words, the association between civic association membership and better mental health might have reflect reverse causation, i.e. the fact that individuals with better mental health status participated in groups, rather than the other way round (participation leading to improved mental health). Second, both our outcome variable and social capital variable were self-reported. If depressed individuals are less likely to rate the trustworthiness of their neighbors in a positive light, this would lead to common method bias. Third, although our questions on social capital were developed on the basis of previous studies, the validity of the individual items (such as inquiring about perceptions of trust) is not established, and further research is required to improve the measurement of community social capital. In addition, the mean score of mental health was lower than that of a previous representative study in Japan (score was 71.6) [18]. There is still a possibility that the raw response rate might cause a potential bias in measuring mental health. Caution therefore is warranted in over-interpreting these findings. Fourth, our overall response rate was low (although not atypical postal surveys of this type). If poor mental health subjects with low social capital tended to refuse to participate in our survey, this selection bias may have had some effect on the resulting association between social capital and mental health. In addition, the majority of participants were older and highly educated. Thus, further research should compare results from different generations (e.g. old and young populations) or socioeconomic status (high and low educational attainment). Fifth, there may be a difference between urban and rural populations [21]. While developing this challenge was beyond the scope of our paper, we consider this as a potentially important issue for further exploration.

Our study also has some strengths. To our knowledge, it is the first study of social capital and mental health in Japan that utilized a nationally representative sample. Our areal unit for measuring neighborhood social capital was based on prior theory that incorporates relevant sociodemographic characteristics of residents. We measured and tested two separate dimensions of social capital – the cognitive and the structural – and we tested them in a multilevel analytical framework to rule out the influence of confounding by compositional factors. Our findings suggest that the concept of community social capital may have relevance for mental health promotion in Japanese society.
